# Animal-Assisted Interventions for the Improvement of Mental Health Outcomes in Higher Education Students: A Systematic Review of Randomised Controlled Trials

**DOI:** 10.3390/ijerph182010768

**Published:** 2021-10-14

**Authors:** Charlotte Parbery-Clark, Marvellas Lubamba, Louise Tanner, Elaine McColl

**Affiliations:** 1 Population Health Sciences Institute, Newcastle University, Newcastle upon Tyne NE1 7RU, UK; lubamba@ualberta.ca (M.L.); louise.tanner@newcastle.ac.uk (L.T.); elaine.mccoll@newcastle.ac.uk (E.M.); 2 Newcastle City Council, Civic Centre, Newcastle upon Tyne NE1 8QH, UK

**Keywords:** animal-assisted interventions, mental health outcomes, stress, anxiety, higher education, systematic review

## Abstract

Background: The aim of this systematic review was to evaluate the effectiveness of Animal-Assisted Interventions (AAIs), particularly Animal-Assisted Therapy (AAT) and Animal-Assisted Activity (AAA), in improving mental health outcomes for students in higher education. The number of students in higher education reporting mental health problems and seeking support from universities’ student support services has risen over recent years. Therefore, providing engaging interventions, such as AAIs, that are accessible to large groups of students are attractive. Methods: MEDLINE, PsycINFO, Embase and Cochrane Library were searched from relative inception to end of April 2020. Additionally, a grey literature search was undertaken. Independent screening, data extraction and risk of bias assessment were completed, with varying percentages, by two reviewers. Results: After de-duplication, 6248 articles were identified of which 11 studies were included in the narrative synthesis. The evidence from randomised controlled trials suggests that AAIs could provide short-term beneficial results for anxiety in students attending higher education but with limited evidence for stress, and inconclusive evidence for depression, well-being and mood. For the non-statistically significant results, the studies either did not include a power calculation or were under-powered. Conclusions: Potential emerging evidence for the short-term benefits of AAI for anxiety, and possibly stress, for students in higher education was found.

## 1. Introduction

Attending higher education commonly represents a major life transition for young people, with it often being the first time living away from the family home, which can bring social, financial and academic stressors [1,2]. The true prevalence of mental health problems for students in higher education is hard to estimate accurately. For example, in the UK, there is a scarcity of large-scale studies being truly representative of the UK student population in higher education or applying a weighted adjustment to accommodate for the lack of representativity [3,4]. However, the number of students in higher education disclosing mental health problems and accessing higher education institutions’ (HEI) support services has risen in recent years [1]. Disclosure and requesting support can result in long waiting lists for more traditional individualised therapy sessions, while stigma around seeking help for mental health and well-being is still present [5,6]. Therefore, a possible solution may be the provision of interventions aimed at reducing stress and anxiety as well as boosting mental health and well-being that are appealing, effective, and accessible to large groups of students [6]. In this respect, part of the solution could be Animal Assisted Interventions (AAIs). 

AAI is an umbrella term that describes the use of various animal species in numerous ways that are beneficial to humans, and includes Animal-Assisted Therapy (AAT), Animal-Assisted Education (AAE), Animal-Assisted Activity (AAA) and more recently, Animal-Assisted Coaching (AAC) [7,8,9]. In summary, AAT is a structured and goal-directed intervention with a specifically trained live animal and is designed to ameliorate socio-emotional, behavioural, cognitive and/or physical functioning [7,8]. AAA is a planned informal interaction with human-animal teams for recreational, motivational and educational opportunities [7,8]. AAE, similarly structured to AAT, focuses on specific educational or academic goals with a professional trained in, and with expertise, in education or a similar field [7,8]. AAC is also similarly structured to AAT and AAE but delivered by licensed coaches focusing on personal growth [8]. AAIs’ popularity have risen over recent years, and they are used in diverse settings such as hospitals, nursing homes, schools and universities [10,11,12,13,14]. Positive interactions with animals have been shown to have beneficial human physiological responses, such as reduction in heart rate, blood pressure, stress hormones (for example, cortisol), and increase in hormones associated with positive emotions (for example, oxytocin) [15,16,17,18]. Evidence has emerged that AAIs, particularly AAT, may be effective in treating various mental health conditions (such as schizophrenia, depression and drug/alcohol addiction), developmental disorders (such as autism-spectrum disorder) and depressive symptoms in individuals with certain neurological conditions (such as dementia) [10,11,14,19]. Furthermore, a recent meta-analysis, involving both children and adults, demonstrated statistically significant improvements in heart rate, self-reported anxiety and stress, but not blood pressure, after AAIs [20]. The discrepancies in findings related to BP may be due to psychological arousal and do not necessarily contradict the stress relieving effects [21]. 

There are numerous theories of why and how AAIs may work. For example, Crossman et al. [6] suggests various theories for how animals may reduce stress including:

- emotional contagion (transmitting the animal’s positive emotions onto humans)
- facilitating social interaction
- opportunities for reinforcement (by partaking in pleasurable activities and experiencing positive emotions)
- evoking expectations that participation will reduce stress (expectancy that the intervention will work)

Beck [22] describes that the human–animal bond is rooted in evolutionary as well as physiological and psychological processes with significant health benefits for both humans and animals. Furthermore, the importance, in the psychosocial model, of social support for health and how social support can function as a buffer against stress are also relevant [23,24]. The animal–human bond can be considered as a type of social relationship, which can offer this type of support. Some individuals may form an animal–human bond more readily than a human-human bond as animals are considered to be indifferent and non-judgemental to an individual’s appearance, social skills or socioeconomic status (SES) [25]. Furthermore, the Biophilia Theory proposes that humans are drawn to interact with animals due to an innate desire to connect with living organisms and nature [25,26]. Additionally, distraction as a cognitive refocus may also contribute, though this research has mostly focused on anxiety and pain whilst awaiting or receiving medical treatment [27]. 

Interestingly, the prevalence of programmes using AAIs at HEIs has increased, for example by 2015 over 900 existed in the USA [6]. In the UK, these types of programmes have also risen in popularity with various forms being implemented, ranging from one-day events to specific sessions [28,29,30,31,32]. Additionally, the evidence-base for using AAIs with this population is growing with persuasive descriptive and anecdotal reports of the benefits [2,6,33,34]. Over recent years, more randomised controlled trials (RCTs) have been published, evaluating the effectiveness of AAIs in respect of various outcomes for students in higher education [35,36,37]. Nonetheless, a lack of completed systematic reviews for AAIs and this specific population exists. Therefore, to our knowledge, no completed systematic reviews were identified that primarily evaluate the effectiveness of AAIs, particularly AAT and AAA, delivered in multiple or single sessions, in improving mental health outcomes for students in higher education, with no age or course restrictions. This systematic review addresses this gap in the literature to help inform HEI providers regarding the potential benefits of AAIs for students’ mental health and well-being, as well as to provide recommendations for future policy, practices and research.

## 2. Aim and Objectives 

The aim of this systematic review was to evaluate the effectiveness of AAIs, particularly AAT and AAA, in improving mental health outcomes for students in higher education. 

The objectives were to:

- systematically search and critically appraise the relevant published and unpublished literature on the effectiveness of AAIs, particularly AAT and AAA, in improving mental health outcomes for this particular population.
- provide evidenced-based recommendations for policy, practice and further research.

## 3. Methods

### 3.1. Protocol and Ethics

A scoping review defined the focus of this systematic review by identifying gaps in the literature. This included searches for published literature in MEDLINE, The Cochrane Library, PsychINFO and Campbell Collaboration, as well as PROSPERO and Joanna Briggs Institute’s Systematic Review Register. The protocol for this systematic review was peer-reviewed and registered on the PROSPERO database on 25 June 2020 (registration number CRD42020186541) [38]. The Preferred Reporting Items for Systematic Reviews and Meta-Analyses (PRISMA) guidelines were followed [39]. Ethics approval was not required. 

### 3.2. Search Strategy

The search strategy was independently peer-reviewed by both an information specialist and an experienced librarian at Newcastle University. The full search strategies are included in Appendix A. The search strategies were not limited by year, study design, language or publication status. MEDLINE, PsycINFO, Embase and Cochrane Library with inclusion of Central Register of Controlled Trials CENTRAL, Cochrane Database of Systematic Reviews and Cochrane Clinical Answers were searched from their relative inception to week three of April/week commencing 27 April 2020. Additionally, an Advanced Google search, using the first four pages due to Google sorting by relevance, during the week ending the 1 May 2020, as well as a further search in the PROSPERO database were completed. To identify additional studies, the reference lists of all full manuscripts meeting eligibility criteria and a “cited in” search using Science Citation Index/Science Citation Index Expanded via Web of Science were reviewed. 

### 3.3. Inclusion and Exclusion Criteria 

A description of the inclusion and exclusion criteria, according to PICOS (Population, Intervention, Comparator, Outcome and Study design), has been provided below and summarised in Table 1 [40]. During study selection, no restrictions for study geographical location, date or language were applied. 

The population was students in higher education with no age, course or location restrictions. Higher education was operationalised as delivered beyond secondary school leading to a degree [46]. Studies that only included students with an already established diagnosis were excluded, as this would have substantially affected the clinical heterogeneity of the studies being compared. Consequently, the intervention’s true effect might have been affected by differences in the population and not the intervention itself, thereby potentially compromising the generalisability of the results [40].

Differences regarding the definitions, corresponding terminology and operating practices of the various types of AAIs can lead to difficulties assessing and comparing the interventions [7,8,9,47,48,49]. AAA and AAT are often used interchangeably in the literature, leading to ambiguity [12,49]. To overcome these identified discrepancies, both AAT and AAA were included. The definitions provided by the International Association of Human-Animal Interaction Organisations (IAHAIO) and the American Veterinary Medical Association (AVMA) to classify the various types of AAI were used [7,8]. In summary, AAT involves a specifically trained live animal in a planned, structured and goal-directed intervention, designed to improve socio-emotional, physical, behavioural and/or cognitive functioning of the individual(s) as part of the treatment process [7,8]. AAT is delivered and/or directed by a trained human professional (from education, health or human services) with specific expertise, and progress is measured/evaluated [7,8]. AAA is a planned informal interaction with trained animal-human teams for recreational, motivational and educational opportunities [7,8]. For the purposes of this systematic review, in accordance with IAHAIO, AAA is goal-orientated [8]. It was anticipated that studies might provide insufficient detail to objectively assess and classify the type of intervention (for example to distinguish clearly between AAT and AAA). Consequently, studies were included if the live animal was called/considered a therapy animal, a therapeutic aim/goal was identified, and the outcomes of interests were evaluated. If the term “therapy animal” had not been used, the authors had to explicitly mention that the animal had, at least, had introductory training and an assessment/evaluation [8,9]. If the intervention was part of a multi-component programme, isolating the effectiveness of the AAT/AAA had to be possible; otherwise, the study was excluded. Additionally, if a study used a stressor, the stressor had to be an aspect of study, training, education or be student specific. An exam or an experimental cognitive test that was used to emulate evaluative testing are examples of included stressors.

Any type of comparator was included, including active intervention, attention control, placebo/sham therapy, usual care/treatment as usual, or alternative active intervention. If there was more than one comparator, one was chosen according to a hierarchy that was established to assess the intervention’s effectiveness [40,50]:

1. control (no-treatment, attention, usual care, or wait-list)
2. validated sham treatment (where known not to be efficacious)
3. other active intervention with known efficacy
4. sham/alternative treatments (where efficacy is unknown)

Specific psychological mental health outcomes, assessed using various established or published standardised measures, before and after the intervention were included. The primary outcomes of effect focused on anxiety and/or stress, using a range of established or published standardised measures, including but not limited to, Perceived Stress Scale (PSS) or State-Trait Anxiety Inventory (STAI) [51,52]. These outcomes were chosen as students in higher education may experience a significant amount of stress [6,53,54,55]. Additionally, anxiety can occur as a reaction to stress, with stress and anxiety being closely linked [56]. Differences in anxiety and/or stress scores from baseline pre-intervention to directly post-intervention and/or final follow-up were included. Secondary outcomes of effect focused on mood/affect, depression and well-being, using a range of published or established standardised measures, including but not limited to, Warwick–Edinburgh Mental Well-being Scale (WEMWBS) or Positive and Negative Affect Schedule (PANAS) [57,58]. The time-point considered to have the biggest potential health benefit was considered as the time-point immediately after the intervention. Thereafter, the next best alternative was the time-point closest to the end of the intervention.

RCTs represent the gold standard for measuring an intervention’s effectiveness with high internal validity [59,60]. Therefore, only RCTs were included. Studies were excluded if allocation to the respective groups was not objectively randomised, for example if randomisation was according to participants’ availability, student number or date of birth with no random component. Any pilot/exploratory studies that met all inclusion criteria and analysed the outcomes of interest were included, unless the full RCT had been reported.

### 3.4. Study Selection

Following the electronic database and grey literature searches, titles and abstracts (*n* = 8036) were de-duplicated. The remaining titles and abstracts (*n* = 6248) were cautiously screened for relevance, erring on over-inclusivity, by the first author (CPC) in Rayyan to remove obvious irrelevant studies or duplicated studies not identified by the automated systems [40,61]. Subsequently, 100% of the articles that were deemed potentially relevant (*n* = 928), were reviewed independently by two reviewers (CPC and ML, a fellow Master’s student) against the pre-specified eligibility criteria using Rayyan [61]. 132 articles were identified as requiring full manuscript review, which was undertaken in full by CPC and 20% by ML, both blinded to the other’s decisions. To identify a random 20% for ML, the titles were arranged alphabetically in Endnote. Subsequently, a random number (*n* = 114) was generated by a true random number generator website [62]. Every fifth article (as 20%) was chosen starting from the 114th article. If any discrepancies arose at any stage, discussion occurred between the two reviewers. If consensus was not achieved, agreement was obtained by discussing with a third reviewer (EM). If the full manuscripts were not available, title/abstract/keywords were reviewed. To meet the eligibility criteria to request an inter-library loan, at least three elements of the PICOS criteria had to be fulfilled. Keywords were identified from Rayyan, Endnote and MeSH analyser [61,63,64].

### 3.5. Data Extraction

A structured Microsoft Excel data extraction form was adapted with permission of Dr. C Marshall after being piloted with two studies. The TIDieR checklist was incorporated as AAI is a complex intervention [65]. The data extraction form included [13,66]:

- study characteristics (such as design, setting and country)
- participants (including eligibility criteria, age, gender, and type of student)
- interventions (for example single/multiple sessions, species of animal, if handler present, duration and frequency of sessions as well as length of programme)
- outcomes (such as the relevant measures used, interpretation, results and time-points for measurements)

Data extraction with rigorous double-checking was primarily undertaken by CPC. ML independently data extracted 18% (*n* = 2 out of 11). The same previously described strategy for addressing disagreements was followed, as required.

### 3.6. Risk of Bias Assessment and Strength of Evidence

A validated tool, Risk of Bias 2.0-revised (RoB2) for individually randomised parallel-group trials, was used for the risk of bias assessment [67]. As this systematic review was aimed to inform a health policy question, the effect of interest was the effect of assignment to AAIs [68]. For each study that reported more than one of the relevant primary or secondary outcomes, a risk of bias assessment was completed for each relevant outcome. For each study that reported multiple time-points for the assessment of outcomes or had more than one comparator, one time-point and one comparator were chosen according to the hierarchies previously described. Where the RoB2 guidance did not cover specific situations found in this review, decision rules were developed and applied in a standardised manner (Appendix B). The RoB2 assessment was completed in full by CPC with 18% (*n* = 2) undertaken independently by ML. The same previously described strategy for addressing disagreements was followed. Authors were contacted to request clarifications or to access missing data and given a two-week period to reply.

A narrative synthesis based on the Economic and Social Research Council (ESRC) Methods Programme was planned [69]. Meta-analysis was not appropriate due to all studies being at high risk of bias for the outcomes of interest, with most having high or some concerns regarding missing data, alongside the substantial clinical heterogeneity. Since meta-analysis was judged to be inappropriate, a harvest plot using vote counting, based on direction of effect, was used with categorisation of the studies by their effect (detrimental, no or beneficial effect) [40]. Effect size or statistical significance were not included for this categorisation as this can be misleading [40]. Vote counting was used for both the primary and secondary outcomes using mean change score for one comparator and one time-point according to the hierarchy previously described. If the outcomes were measured immediately after the intervention, as well as, at an additional time with a stressor, the former was only included for the vote counting. A set of decision criteria were created for interventions with a stressor and those without to standardise interpretation of the expected response to the intervention (Appendix B).

The quality and relevance of evidence was appraised using the ’Weight of Evidence’ approach [70] and described in the guidance on narrative synthesis for the ESRC guidance [69]. In accordance with the ESRC guidance, trustworthiness was measured by Jadad’s scale [69,71].

## 4. Results

### 4.1. Study Selection

**Figure 1:**
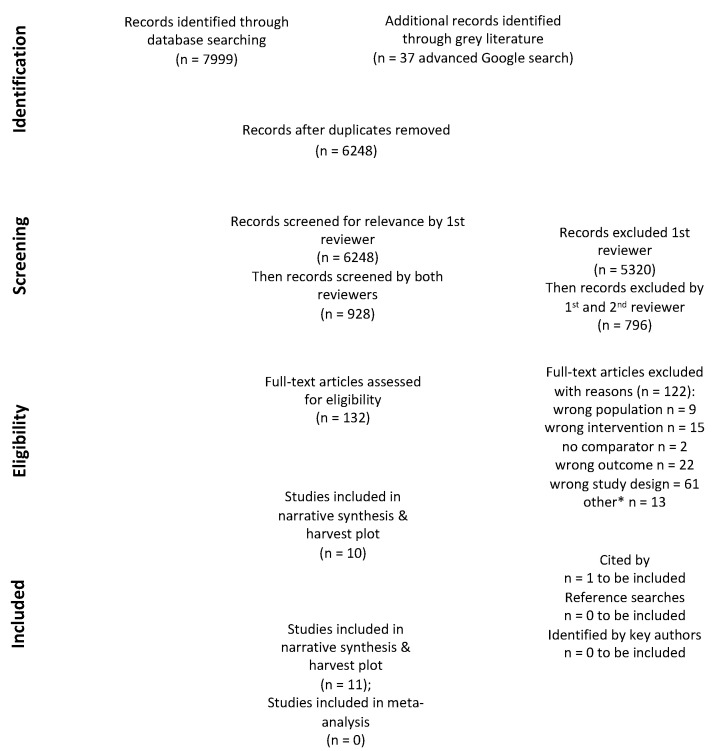
Adapted preferred reporting items for systematic reviews and meta-analysis (PRISMA) diagram adapted from Moher et al. [39]; * other included not available due to COVID-19 (*n* = 2), did not meet criteria for requesting inter-library loan (*n* = 10) & ongoing trial (*n* = 1).

Eleven articles, describing eleven studies, met the inclusion criteria. The PRISMA flowchart is displayed in Figure 1. Ten studies were journal articles and therefore classed as published literature [35,37,72,73,74,75,76,77,78,79]. One study was a dissertation for partial fulfilment of PhD and classed as grey literature [80]. All 11 studies were individually randomised parallel-group trials. Of these, three were described as pilot or exploratory studies but nonetheless presented data on interventions’ effectiveness [37,75,80]. The excluded full text published studies (with reasons) are listed in Appendix C.

### 4.2. Study Characteristics

Six studies were conducted in USA [72,75,76,77,79,80], three in Canada [35,73,78], one in Scotland [37], and one in Austria [74]. Sample sizes (for those randomised) across all included studies varied from 20 to 357 students.

#### 4.2.1. Population

For the majority (9 studies), the participants were university students [35,37,72,73,76,77,78,79,80]. Seven studies reported the characteristics according to the recruited sample size [35,37,72,73,76,77,80] and three for the analysed sample size [74,78,79]; one reported the characteristics of the students attending the degree programme from which the participants were recruited and not the sample’s characteristics (this study is excluded from the descriptive statistics below) [75].

Ten studies reported gender with the majority (ranging from 57% to 85%) of participants being female [35,37,72,73,74,76,77,78,79,80]. The most common age bracket was 20-year-olds and under, followed by 21 to 25-year-olds. Type of student was clearly reported in five studies [35,73,76,77,79]. Of those, 80% were undergraduates [35,73,76,77]. The students’ year of academic studies was fully reported in two studies [35,74]. Five studies reported ethnicity [35,73,76,77,80]. None of the studies reported health status or socio-economic status (SES). The participants studied a range of course subjects (such as psychology and nursing). Three studies collected the potential confounder of pet ownership [37,72,78], and one reported experience with the animals employed in the intervention (horses) [80].

#### 4.2.2. Intervention

Ten studies employed dogs [35,37,72,73,74,75,76,77,78,79] and one employed horses [80]. Group sessions were the most common format: seven studies implemented this session type [35,37,72,74,76,77,78], whilst two implemented individual sessions [73,80]. A further two studies implemented either a combination of group and individual sessions [75] or the format of sessions was not clearly reported [79]. The student to dog ratio in the group sessions was clearly reported in two studies [35,37] and implied in three [74,76,77], ranging from 3 to 4-students-per-dog to 12 to 14-students-per-dog.

The presence of a handler during the sessions was clearly reported in seven studies [35,37,72,74,78,79,80]. Nine studies allowed free interaction with the animals [35,37,72,73,75,76,77,78,79] and two used a structured format [74,80]. Most interventions (*n* = 8) corresponded to the definition of AAA described in 3.3 [37,72,73,75,76,77,78,79]. Two studies were classified as AAT [35,74] and one combined AAT and AAE together [80]. These classifications were mostly from an objective assessment of the description provided vis-à-vis the definitions outlined in 3.3. If insufficient detail for an objective assessment, the classification used by the primary authors was kept [35]. Length of intervention ranged from unspecified to 90 min, with the modal length being between 10 to 20 min. Seven studies used a single session [35,37,72,73,78,79,80]. Four studies used multiple sessions [74,75,76,77]; of these, two used once-weekly sessions for four weeks [76,77]; one implemented three sessions with non-reporting of the time interval between sessions [74]; and one allowed various lengths and frequencies over a 15 to 16-week period at the participant’s choice [75]. None of the studies reported monitoring or measuring the intervention’s fidelity. The theoretical frameworks were clearly stated in three studies [35,37,80]. More information regarding the theoretical frameworks is included in Appendix D.

#### 4.2.3. Outcomes

The primary outcomes were self-reported anxiety and stress measured before and after the intervention. Seven studies measured self-reported anxiety [37,74,75,76,77,79,80] with most using the State-Trait Anxiety Inventory (STAI), apart from one study which used the Hospital Anxiety and Depression Scale (HADS) [75]. Two studies measured stress using the Perceived Stress Scale (PSS) on the original 5-point Likert scale [35,78].

Regarding the secondary outcomes, two studies measured depression [75,77] using the HADS-depression subscale and Beck Depression Inventory II (BDI II), respectively. Five studies measured mood/affect, with most using Positive and Negative Affect Schedule (PANAS) [72,73,76,78] and one used the University of Wales Institute of Science and Technology Mood Adjective Checklist (UMACL) [37]. Well-being was measured in three studies [35,37,78] using various tools.

The timing of outcome measurement after the intervention varied substantially between studies (with some reporting multiple time-points outlined in Table 2) and encompassed: immediately after the intervention without a stressor (*n* = 6); after the intervention but before an exam (*n* = 2); after the intervention and an experimental stressor (*n* = 4); within 24 h of the intervention (*n* = 1); 2 weeks after the intervention (*n* = 1); up to 1 month after the intervention (*n* = 1); up to 15–16 weeks since the start of the intervention (*n* = 1).

Regarding adverse events reporting, Hall [75] described ten individuals who reported increased anxiety and stress due to being in the control group and unable to participate with the animal. These participants were subsequently removed from the study to allow interaction. No other adverse events were reported. None of the studies specifically reported any adverse events for the animals involved.

Characteristics are summarised in Table 2.

### 4.3. Risk of Bias Assessment

**Figure 2:**
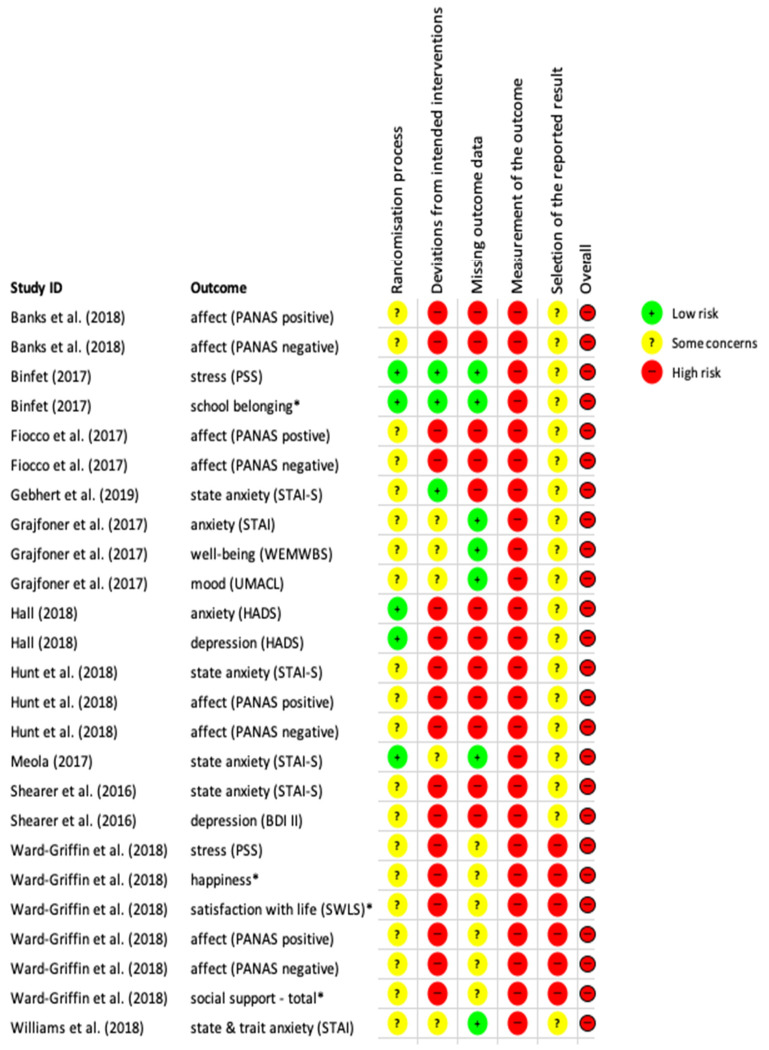
Risk of bias assessment (RoB2 assessment) for all included studies on the outcomes of interest; * considered as a proxy for well-being.

Figure 2 summarises the risk of bias for each domain with an overall assessment for each study for all the relevant outcomes. All studies were at overall high risk of bias for the outcomes of interest as susceptible to high risk of bias in the domain “measurement of the outcome”. This was mainly due to the nature of the intervention as participants could not be blinded and the measures were self-reported, therefore were not objective.

### 4.4. Strength of Evidence 

The quality and relevance of evidence was appraised using the ’Weight of Evidence’ approach [70] and described in the guidance on narrative synthesis for the ESRC guidance [69]. The strength of evidence of the included studies is summarised in Table 3. One study had high overall weight [74], eight [35,37,73,75,76,78,79,80] had medium overall weight and two had low overall weight [72,77].

### 4.5. Narrative Synthesis: Interventions’ Effect

As all the studies were at high risk of bias for the outcomes of interest, with most having high or some concerns regarding missing data, alongside the substantial clinical heterogeneity, a meta-analysis was not appropriate as it was unlikely to provide meaningful results.

All the included measurement scales provided continuous data; results were presented in various formats such as pre- and post-values, mean change or adjusted estimates of the intervention’s effects (for example, using analysis of covariance (ANCOVA) with baseline measurements included as a covariate). Appendix D provides a summary of the studies’ results with mean change (post-scores minus pre-scores) as the common parameter using the most immediate measurement of outcomes after the intervention (or the next best alternative).

#### 4.5.1. Primary Outcomes: Anxiety

Seven studies reported the intervention’s effect on self-reported anxiety levels [37,74,75,76,77,79,80]. Six studies employed dogs [37,74,75,76,77,79] and one employed horses [80]. Four studies used group sessions [37,74,76,77], one used individual sessions [80], one offered both group and individual sessions [75] and one did not explicitly state the type of sessions but was inferred to be individual [79]. Session length varied and included student’s choice to sessions lasting 12, 20 and up to 60 min. Three studies offered a single session [37,79,80], two offered four sessions (once/week) [76,77], one varied depending on students’ choice [75] and one offered three consecutive sessions but did not report the time interval between sessions [74]. Most interventions were consistent with AAA [37,75,76,77,79] and of these studies (*n* = 5), two included, alongside the dogs, interactive games, icebreakers and snacks for the participants/dogs [76,77]. Furthermore, Gebhart et al. [74] offered AAT and Meola [80] offered tailored and structured individual sessions called equine-assisted learning supervision (EALS). The latter incorporated both educational and therapeutic goals, therefore combining AAE and AAT.

Six studies tested outcomes without a stressor, with four showing a statistically significant improvement in favour of the AAI [37,74,75,77]. Four studies tested the outcomes immediately after the intervention without a stressor, with three showing a statistically significant reduction in favour of the AAI [37,74,77] and one showing a non-statistically significant reduction but did not include a power calculation [76]. For the two studies which tested the outcome after a longer time interval, Meola [80] found a non-statistically significant reduction in anxiety when measured up to one month after the intervention but this study was under-powered. Hall [75] found a statistically significant reduction in the post-intervention scores 15–16 weeks after the intervention had started.

Four studies involved a stressor: two assessed the outcomes after the intervention but prior to an exam [74,79] and two assessed the outcomes after an experimental cognitive test (WAIS-IV IQ test) which was designed to imitate evaluative testing that occurs in higher education [76,77]. Two studies found no statistical difference [74,77] but a power calculation was not included. Hunt et al. [76], despite no significant effect of condition on anxiety after a stressor, still undertook paired comparisons which showed a statistically significant higher level of anxiety in the AAI group compared to the control group. Williams et al. [79] showed an increase in anxiety for both groups, after the intervention but before an exam, which one might therefore expect. However, the control group had statistically higher anxiety levels than the AAI group.

**Figure 3:**
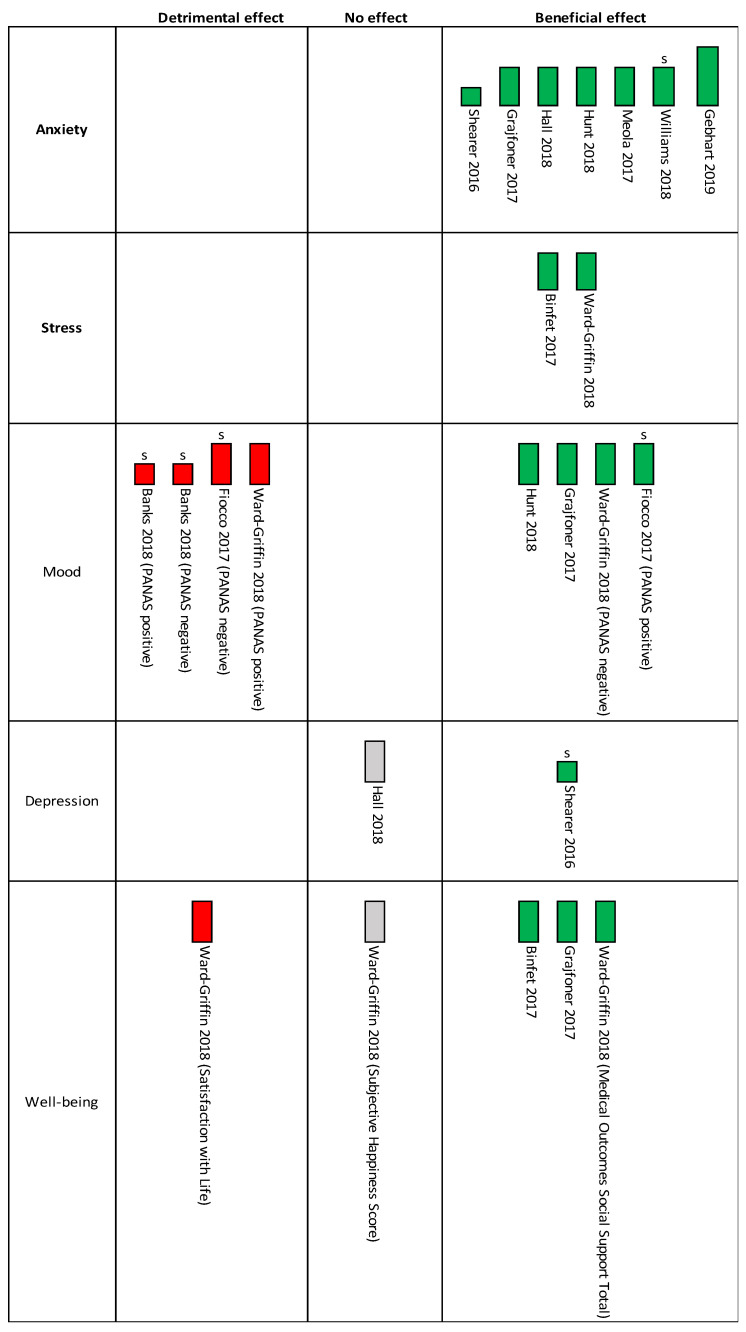
Harvest plot for the primary & secondary outcomes for one comparator & one time-point as described in the methods. s stands for stressor; height of bars relate to overall strength of evidence (tallest = high; shortest = low).

Overall risk of bias was high for all seven studies regarding the outcomes of interest. Regarding overall strength of evidence, one study had high [74], one had low [77] and the remaining five had medium strength. Using vote counting, according to direction of effect and not statistical significance as described in the methods, all seven studies showed a beneficial effect in favour of the intervention compared to the comparator (demonstrated in harvest plot in Figure 3).

#### 4.5.2. Primary Outcome: Stress

Two studies, using single group sessions with dogs, measured self-reported stress [35,78]. Binfet [35] described the intervention as AAT whilst the intervention in Ward-Griffin et al. [78] was consistent with AAA. The sessions lasted varying amounts of time from 20 min [35] to up to 90 min (but on average 30 min) [78]. Neither study used a stressor but one study [78] occurred during mid-term exam season. Despite the differences between the two studies, both showed a statistically significant reduction in stress when measured within 24 h of the intervention. This effect was not sustained at the two-week follow-up for the one study that included longer follow-up [35]. Therefore, these studies showed cautious preliminary evidence of a short-term, statistically significant, beneficial effect on stress, using AAIs, with students at Canadian universities. However, with such a small number of studies (with both being at high risk of bias for the outcomes of interest), caution is needed regarding generalisability to other countries and settings.

#### 4.5.3. Secondary Outcomes: Depression, Mood/Affect and Well-Being

The evidence for depression is only based on two studies with different study characteristics and with mixed results [75,77]. One study, with low strength of evidence, showed a non-statistically significant beneficial effect on depression (with a stressor) [77] but we are unable to state if this is the true effect as the study could have been underpowered. The other study showed a reduction in depression scores for both intervention and comparator but by a larger degree for the control (without a stressor) [75]. However, caution is required due to the data’s distribution (skewed) and how the results were provided (mean) personal communication [81].

The evidence for mood is mixed with five studies measuring this outcome [37,72,73,76,78]. A non-statistically significant detrimental effect appeared to occur particularly when a stressor is applied. Of the two studies that showed statistically significant beneficial results without a stressor, this result had not lasted by the 4th session for one study. Where a non-statistical significance was shown, a power calculation was not included. Therefore, the studies may have been underpowered to demonstrate the true effect (which may or may not be similar to the results explored here). Further investigation is required before any conclusions can be made for this outcome.

Three studies measured well-being using various tools (for example, Sense of Belonging in School, Warwick–Edinburgh Mental Well-being Scale (WEMWBS), Satisfaction with Life Scale (SWLS), Subjective Happiness Scale and Medical Outcomes Study Social Support) [35,37,78]. Overall, tentative but mostly beneficial effects were found in varying measures of well-being when measured immediately after or within 24 h of the AAI. Where a non-statistically significant effect was found, the outcomes were measured within 24 h of the intervention during mid-term exam season. Additionally, no power calculation had been included for the non-statistically significant findings, and therefore, distinguishing between true “no effect” or being underpowered was not possible.

## 5. Discussion

### 5.1. Statement of Principal Findings

This systematic review included 11 RCT assessing AAIs on mental health outcomes for students attending higher education in a variety of settings and countries. The evidence suggests that AAIs could provide short-term beneficial results for anxiety in students attending higher education. There is limited evidence for stress, and inconclusive evidence for depression, well-being and mood. These results are from studies at high risk of bias for the outcomes of interest with mostly medium strength of evidence.

### 5.2. Strengths and Weaknesses of the Review

The strengths of this review include a comprehensive search strategy incorporating both grey and published literature. Additionally, independent conduct of screening, data extraction and risk of bias with inclusion of a third reviewer to resolve any discrepancies were incorporated. This reduced the introduction of random and systematic errors [82]. Development of decision rules, where required, increased transparency and rigor. Furthermore, this review combined RCTs, which represent the gold standard study design for evaluating a causal relationship and for measuring an intervention’s effectiveness [83]. A meta-analysis was not appropriate for reasons already described; therefore, vote counting, based on direction of effect and not statistical significance, was employed. Vote counting using statistical significance can be misleading especially in studies where no power calculation was reported and no significance was found [40]. The lack of statistically significant effect may either be due to the study being underpowered or may reflect a true lack of effect [40]. However, when using vote counting on direction of effect without considering statistical significance, the effect seen may be due to chance. Additionally, vote counting methods are unable to provide a precise estimate of the overall effect size.

Due to COVID-19, some resources were inaccessible (list included in Appendix C). Additionally, a pragmatic approach to the Advanced Google search was incorporated using only primary outcomes. Well-being is a particularly broad concept. Proxies that were strongly related to well-being were included but may not have been an exhaustive list. Therefore, some articles that could have met the eligibility criteria may have been missed. A percentage above a minimum for double screening, data extraction and risk of bias assessment was chosen due to resource and time constraints, which represents a limitation. Using the strength of evidence approach, overall weight was influenced mainly by how well the authors described randomisation and/or presence of withdrawals/missing data as all the studies were relevant RCTs where double blinding was difficult. Where not reported, concluding whether these elements simply had not occurred or had occurred but not described due to reasons (such as word-count limits) was impossible, unless clarified by the authors through correspondence. Furthermore, different tools to assess strength of evidence or risk of bias may have produced different findings.

### 5.3. Strengths and Weaknesses of the Studies

The limitations of the primary evidence reviewed included variable levels of descriptive reporting in the included studies, such as participant characteristics, delivery of the intervention and theory of change. For example, the descriptions of the included AAIs varied considerably from a high level of detail to only a single sentence or short paragraph. Therefore, providing conclusions about whether certain types of AAIs were more effective that others or to isolate the “active ingredient” of effective AAIs was not possible. Most of the participants were females, ranging from 57% to 85%, and authors rarely commented on how well the sample population represented the target population. Furthermore, all the participants volunteered to participate and, therefore, may not be representative of the target population [84]. For example, those volunteering (and therefore, self-selecting) may be more motivated and/or have a different mental health status than those who did not [84,85]. Additionally, individuals who are afraid, allergic or have a medical condition precluding participation with animals are unlikely to have volunteered. Therefore, building a comprehensive picture of the type of individual attending higher education, who would benefit from AAIs was challenging. These reporting issues meant that assessing the generalisability of the results was also problematic.

The outcomes reviewed were self-reported and can be subject to unconscious and/or conscious ascertainment bias. With this type of intervention, blinding of participants or individuals delivering the intervention was difficult, if not impossible. This resulted in all the studies having an overall high risk of bias for the outcomes of interest for this systematic review. Additionally, participant expectancy bias could have been introduced, which was highlighted in Williams et al. [79] as 90% of the control group sampled (*n* = 15) stated they thought an interaction with the dog would have reduced their stress prior to an exam. Despite these limitations, capturing how an individual feels after an intervention/comparator is important. To aid corroboration and confidence with self-reported results, triangulation would be beneficial such as with objective measures (for example, physiological outcomes) and/or blinded behavioural observations [86]. Indeed, some of the studies reported physiological outcomes but were beyond the scope of this systematic review, which is a limitation.

Furthermore, for the outcomes where no statistical difference was found, either no power calculation was included, or the study was underpowered. In those studies, no definitive conclusion can be derived about the effectiveness of that particular intervention for that specific outcome as it is not possible to state if the non-statistically significant difference was the true effect or not [87].

### 5.4. Study Meaning: Possible Mechanism and Implications for Policymakers

The Fogg Behaviour Model (FBM) is a theory of change model that could be considered for AAIs and student engagement to improve mental health outcomes [88]. In summary, FBM requires three elements for the intended behaviour of student engagement with AAIs to occur: motivation, ability and triggers [88]. Triggers promote the intended behaviours and may be achieved by advertisement/promotion of the sessions [88]. Motivation for attendance is proposed as the animals’ presence addressing three core motivators: (1) hope of an experience that is likely to be (2) pleasurable and (3) socially acceptable for the majority [88]. Finally, to optimise the students’ ability to participate in this intended behaviour, the model’s six elements of simplicity should be addressed [88]:

1. time (sessions to be short)
2. money (sessions to be cost-neutral for students)
3. physical effort (sessions to be offered in an accessible location)
4. brain cycles (process by which to attend the sessions should be easy)
5. social acceptance (as offered by activities with animals)
6. routine (regular sessions to be offered)

For policymakers considering implementing AAIs in a higher educational setting, a logic model should be developed alongside the intervention to assist in clarifying the active ingredients and causal assumptions [89]. Key stakeholders, such as students from varying backgrounds, staff from student support services and animal/handler teams, should be involved during the design stage. A formative evaluation, including both process and implementation assessments, with a mixed-methods pilot study would be a useful first step [89,90,91]. The feasibility, acceptability and fidelity of the AAI in the target population can therefore be assessed with adaptation, if required. Thereafter, a summative evaluation, using a larger mixed-methods RCT, evaluating effectiveness, with a nested process evaluation, would be recommended [90].

### 5.5. Future Research Recommendations

Further research recommendations are detailed in Box 1. Particularly, the overall reporting quality by authors should be improved with facilitation from journals. For example, authors should provide enough information to allow replication of the interventions or expansion of the existing research [65]. These steps will help identify the effective or ineffective interventions and facilitate further evaluation of the active ingredients. Furthermore, potentially conflicting evidence, albeit not statistically significant, is present for mood which needs further evaluation to ensure that AAIs do not have unintended negative consequences [92]. Additionally, including animal welfare and economic evaluations are important to help secure support and funding from commissioners.

Box 1 **The recommendations for future research for AAIs.**

- Use of standardised and internationally recognised definitions when describing AAIs
- Use of sample sizes that provide adequate power
- Clarity regarding the randomisation procedure (including description of allocation, and whether concealed allocation occurred) and provision of an adequate description of the participants’ characteristics separated by group
- Clear reporting of the participants’ flow through the trial with reasons for any missing data for each respective group and at each time-point
- Use of explicit comparators to establish the relative effects of the co-interventions (e.g., appropriate attention controls)
- Adequate descriptions of the interventions implemented to facilitate replication
- Clear reporting of the outcome measurement procedure (particularly when multiple time-points or stressors are present), including any adaptations made to the scales used
- Provision of access to publicly available pre-specified statistical analysis plans by authors, including justification for choice of target differences
- Clear reporting of adverse events for both humans and animals

## 6. Conclusions

Animal-assisted interventions (AAIs) were considered as potential interventions to help improve mental health and well-being of students in higher education who are willing to and can engage with animals. The pooled evidence suggests that AAIs could provide short-term beneficial results for anxiety, and possibly stress, in this population, known to be at risk of mental health issues. However, caution is required as these results were from studies at high risk of bias for the outcomes of interest for this systematic review with mostly medium strength evidence, and in various cultural settings. Subsequent implementation of AAIs in this setting requires both formative and summative evaluation to measure both the intended and unintended consequences. Furthermore, consideration of alternatives for students unable to participate due to fear of animals or medical contraindications is recommended to prevent widening of health inequalities.

## Figures and Tables

**Table 1:** Summary of inclusion & exclusion criteria with rationale [7,8,40,41,42,43,44,45,46].

Inclusion	Exclusion
**Population:** Students in higher education (defined as post-secondary education leading to a degree). OR A description of an equivalent/use of terms known to be associated with higher education. Rationale: Definition of higher education and represents a population exposed to significant stress. If stressor present, had to be an aspect of study, training, education or be student-specific. Rationale: student-specific stressor.	**Population:** Students who all have an established diagnosed condition/disorder (such as autism or ADHD). Rationale: could substantially affect the clinical heterogeneity of the populations being compared.
**Intervention:** AAI—particularly AAT and AAA. OR Live animal considered/called a therapy animal (OR animal had training AND assessment or evaluation/certification), a therapeutic goal/aim was identified, and the outcomes of interest were evaluated. Rationale: Key elements of AAI (including AAT and AAA); evaluation of relevant outcomes was required to assess results.	**Intervention:** Not involving a live animal. Rationale: AAIs involve live animals. Participants’ own pets/companion/support/assistance/service animals. Rationale: Likely to represent potential confounders/effect modifiers and not consistent with definitions of AAI, AAT or AAA.
**Comparator:** A comparison group required. Rationale: comparators are required to evaluate intervention’s effectiveness.	**Comparator:** No comparator.
**Outcome:** Psychological using published or established standardised measures: **Primary outcomes:** effect on anxiety and/or stress. **Secondary outcomes:** effect on depression, mood/affect and well-being. Rationale: Represent important measures of mental health and well-being.	**Outcome:** Physiological. Rationale: Often used as proxy measures for psychological states but not directly related to psychological outcomes. Educational/or academic. Rationale: Focus is on mental health and well-being, not performance.
**Study:** RCT and other types of randomisation. Rationale: RCT represents gold standard for measuring effectiveness.	**Study:** all non-randomised. Rationale: prone to effects of confounding & to ensure feasibility of review due to time/resources constraints.

**Table 2:** Characteristics of included studies ordered alphabetically with relevant outcome measures (NR = not reported; SD = standard deviation).

First Author, Year & Country	Participants	Intervention			Comparator	Outcomes	
	Characteristics	Theoretical Framework Articulated	Description of Intervention	Type of AAI & Delivery		Tools Used	Time-Points
Banks [72] 2018 USA	University students with some recruited from psychology department (76.8% female) Mean age 20.05 (SD 3.38) Year of study, type of graduate, ethnicity, health status or SES NR	No	Group (free interaction) with as many dogs as wanted (student: dog ratio NR) for 10 min single session during mid-term exam week Various breeds (e.g., Beagle, Golden Retrievers, German Shepherds)	AAA Handler	No-treatment control	PANAS—positive & negative	Pre & post stressor (SART & letter/ pattern comparison)
Binfet [35] 2017 Canada	First-year university undergraduate students taking psychology classes (78% female) Mean age 18.85 (SD 2.65) Ethnicity: 57% Caucasian; 15% Chinese; 9% Mixed-Race Health status or SES NR	Yes	Group (free interaction) 3–4 student: 1 therapy dog/handler for 20 min single session Various breeds including pure-bred & mixed (which breeds NR)	AAT * Handler	No-treatment control (“business as usual” control = studying)	PSS Sense of Belonging in School	Pre, post, & then follow-up after 2 weeks
Fiocco [73] 2017 Canada	Undergraduate university students (77.1% female) Mean age 21.02 (SD 5.5) Ethnicity: 37.7% Caucasian; 8.2% Black/African American; 54.1% Other Year of study, health status or SES NR	Partial	Individual (free interaction as long as participant remained seated) with a dog for 10 min single session Various breeds of different ages & sizes/breed (e.g., Irish Setter, Schnoodle, Greyhound, King Charles Spaniel)	AAA Unclear if handler present	No-treatment control (sitting for 10 min)	PANAS—positive & negative	Pre, post AAA & then after stressor (PASAT)
Gebhart [74] 2019 Austria	First-year students at nursing school (77% female) Median age 20 (IQR 19–22) Type of graduate not clearly specified; health status, ethnicity or SES NR	Partial	Group interaction (structured with different tasks, playing & interacting with dogs) student: dog/handler ratio implied 3 students: 1 therapy dogs/handler for 45–60 min for 3 sessions (time interval between sessions NR)	AAT Handler	No-treatment control unstructured free hour; music therapy (body percussion) & mandala painting	STAI-S	Pre & post normal day Pre & post exam day
Grajfoner [37] 2017 Scotland	University students (64.4.% female) Mean age 21.6 (SD 3.4) Year of study, type of graduate, health status, ethnicity or SES NR	Yes	Group (free interaction) ~6 students: 1 dog/handler ratio for 20 min single session Various breeds (e.g., Labrador, Lhasa Apso, Golden Retriever)	AAA Handler	Handler only (HO) & dog only (DO) (handler present; no interaction)	STAI WEMWBS UMACL	Pre & post
Hall [75] 2018 USA	Level 2 community college associate degree nursing programme Gender, age, year of study, type of graduate or health status, ethnicity or SES NR (only demographics of the course)	Partial	Mix of group or individual session (free interaction) with numerous opportunities to interact with dog. Dog on campus minimum twice a week, visited students at various locations & on exam days for 30 min pre-exams. Intensity, length & frequency of sessions NR Standard Poodle	AAA Handler (unclear if always present)	No-treatment control	HADs—anxiety & depression	Pre & post (which is after 15–16 weeks from start of intervention)
Hunt [76] 2018 USA	Undergraduate students enrolled in psychology courses (74% female) Mean age 19.3 (SD NR) & ≥18 yrs old Ethnicity: 52% non-Hispanic White; 27% Asian/Asian-American; 9% Hispanic/Latino; 5% Black/African American, 4% Multiracial, 2% Indian; 1% Arab Year of study, health status or SES NR	Partial	Group interaction (free interaction) with a dog plus interactive games, icebreakers & snacks student: dog ratio not clearly stated but implied 12–14 students: 1 dog of unclear length for once/week for 4 sessions Golden Retriever	AAA Unclear if handler present	No-treatment control; mindfulness training alone; yoga alone; or mindfulness training with yoga	STAI-S PANAS—positive & negative	Pre & post every session & once 1–3 weeks after completion of AAA with stressor (WAIS-IV IQ test)
Meola [80] 2017 USA	University students enrolled on accredited counselling program (85% female) Mean age 30.8 (SD NR) Ethnicity: 85.7% Caucasian; 9.5% African American Year of study, type of graduate (some Masters & PhD but NR for all), health status, or SES NR	Yes	Individual (structured & tailored) equine-assisted learning supervision (EALS) session with a horse with 3 different activities: for 1-h single session	AAT/AAE Handler (who was also an instructor/ facilitator)	No-treatment control	STAI-S	Pre & then post up to one month after intervention
Shearer [77] 2016 USA	Undergraduate university students in psychology courses (57% female) Ethnicity: 43% Asian; 41% Caucasian; 7% Hispanic; 3% African American; 3% Other; 1% Native American; 1% Pacific Islander; 1% unidentified. Year of study, health status, age or SES NR	Partial	Group session (free interaction) with a dog plus games & snacks students: dog ratio not clearly specified but implied 12–13 students: 1 dog for 1 h/week for 4 weeks	AAA Facilitator	No-treatment control (added in 2nd phase) or mindfulness meditation	STAI-S BDI II	Pre & then post each session for 4 weeks & then once 1–2 weeks after completion of AAA with stressor (WAIS-IV)
Ward-Griffin [78] 2018 Canada	University students enrolled in introductory psychology classes (78% female) Mean age 19.4 (SD 3.73) 45.5% first-year students; others NR Type of graduate, health status, ethnicity or SES NR	Partial	Group session (free interaction) with dogs. student to dog ratio not clearly specified with 7–12 dogs present with handlers for up to 90 min single session during mid-term exam season (on average participants spent 30 min in the space)	AAA Handler	Wait-list control	PSS PANAS—positive & negative SWLS Subjective Happiness Scale Medical Outcomes Study Social Support Scale	Pre & within 24 h of AAA
Williams [79] 2018 USA	University graduate students in pharmacy/physical therapy (63.2% female) Mean age 24.42 (SD NR) Year of study, health status, ethnicity, or SES NR	Partial	Not clearly specified but inferred as individual free interaction (as long as no active running & playing) with a dog for 12 min single session prior to exam	AAA Handler	No-treatment control (“business as usual” control = quiet time studying)	STAI-S & T	Pre & post (but before an exam)

* as described by the authors; limited information to provide an objective assessment of type of AAI. Abbreviations used: AAA: Animal-Assisted Activity; AAE: Animal-Assisted Education; AAI: Animal-Assisted Intervention; AAT: Animal-Assisted Therapy; BDI-II: Beck Depression Inventory II; HADS: Hospital Anxiety and Depression Scale; IQR: interquartile range; NR: not reported; PANAS: Positive and Negative Affect Schedule; PASAT: Paced Auditory Serial Addition Task; PSS: Perceived Stress Scale; SART: Sustained Attention to Response Task; SES: socioeconomic status; SD: standard deviation; STAI: State-Trait Anxiety Inventory; STAI-S: State-Trait Anxiety Inventory state anxiety; STAI-T: State-Trait Anxiety Inventory trait anxiety; SWLS: Satisfaction with Life Score; WAIS-IV IQ: Wechsler Adult Intelligence Scale-IV.

**Table 3:** The strength of evidence for the included studies in accordance with the ’Weight of Evidence’ approach [70].

Study	Trustworthiness (A)	Appropriateness (B)	Relevance (C)	Overall Weight (D)
Banks et al. [72]	Low	High	High	Low
Binfet [35]	Medium	High	High	Medium
Fiocco et al. [73]	Medium	High	High	Medium
Gebhart et al. [74]	High	High	High	High
Grajfoner et al. [37]	Medium	High	High	Medium
Hall [75]	Medium	High	High	Medium
Hunt et al. [76]	Medium	High	High	Medium
Meola [80]	Medium	High	High	Medium
Shearer et al. [77]	Low	High	High	Low
Ward-Griffin et al. [78]	Medium	High	High	Medium
Williams et al. [79]	Medium	High	High	Medium

## Data Availability

Not applicable.

## Abbreviations

AAA	Animal-Assisted Activity
AAC	Animal-Assisted Coaching
AAE	Animal-Assisted Education
AAI	Animal-Assisted Intervention
AAT	Animal-Assisted Therapy
ADHD	Attention Deficit Hyperactivity Disorder
ANOVA	analysis of variance
ANCOVA	analysis of covariance
BDI-II	Beck Depression Inventory II
DO	dog only
EALS	equine-assisted learning supervision
EPPI	Evidence for Policy and Practice Information
ESRC	Economic and Social Research Council
FBM	Fogg Behavioural Model
F/up	follow-up
HADS	Hospital Anxiety and Depression Scale
HEI	higher education institute
HO	handler only
IAHAIO	International Association of Human-Animal Interaction Organizations
IQR	interquartile range
IRB	Institutional Review Boards
MANCOVA	multivariate analysis of covariance
MANOVA	multivariate analysis of variance
MeSH	Medical Subject Headings
MOS Social Support Scale	Medical Outcomes Study Social Support Scale
NR	not reported
NS	not significant
PANAS	Positive and Negative Affect Schedule
PASAT	Paced Auditory Serial Addition Task
PICOS	Population, Intervention, Comparator, Outcome, Study design
PSS	Perceived Stress Scale
RCT	randomised controlled trial
SART	Sustained Attention to Response Task
SD	standard deviation
SES	socioeconomic status
SSS	student support services
STAI	State-Trait Anxiety Inventory
SWLS	Satisfaction with Life Score
TIDieR	template for intervention description and replication
UMACL	University of Wales Institute of Science & Technology Mood Adjective Checklist
UWIST	University of Wales Institute of Science & Technology
WAIQ Scale-IV	Wechsler Adult Intelligence Scale-IV
WEMWBS	Warwick-Edinburgh Mental Well-being Scale
Yrs	years

## Appendix A

**Search Strategies**

The search strategy was independently peer-reviewed by both an information specialist and an experienced librarian at Newcastle University.

**MEDLINE through OVID (from 1946 to 28 April 2020):**

(1) [Student$ or pupil$ or undergrad$ or postgrad$ or graduat$ or freshm?n* or sophomor$ or junior$ or senior$ or learner$ or scholar$ or apprentic$ or classmate$].ti.kw.ab
(2) [junior$ or senior$] adj1 year$].ti,kw,ab.
(3) Exp students/
(4) [colleg$ or universit$ or school$ or conservator$ or classroom$ or apprenticeship$ or facult$].ti,kw,ab.
(5) [[educat$ or graduat$ or undergrad$ or academ$ or junior$ or senior$ or postsecondary$ or ‘post secondary$’] adj1 [school$ or colleg$ or universit$ or institut$ or setting$ or facult$ or establish$ or program$]].ti,kw,ab.
(6) [[seminar$ or lectur$] adj1 [room$ or theatre$]].ti,kw,ab
(7) Exp schools/
(8) Exp “internship and residency”/
(9) Exp faculty/
(10) Exp nursing faculty practice/
(11) Exp education, nonprofessional/
(12) Exp education, predental/
(13) Exp education, premedical/
(14) Exp education, professional/
(15) Exp inservice training/
(16) Exp international educational exchange/
(17) Exp “academies and institutes”/
(18) 1 or 2 or 3 or 4 or 5 or 6 or 7 or 8 or 9 or 10 or 11 or 12 or 13 or 14 or 15 or 16 or 17
(19) “human$ animal$ interact$’.ti,ab,kw.
(20) Exp bonding, human-pet/
(21) ‘human$ animal$ bond$’.ti,ab,kw.
(22) Exp animal assisted therapy/
(23) [[animal$ or pet$ or dog$ or canine$ or hound$ or pooch$ or pup$ or cat$ or feline$ or kitt$ or equine$ or horse$ or hippo$ or pony$ or foal$ or riding$ or ‘guinea$ pig$’ or rabbit$ or bunn$ or ferret$ or hamster$ or rodent$ or mammal$ or bird$ or cow$ or pig$ or sheep$ or lamb$ or dolphin$ or aquatic$ or fish$ or marine$ or reptile$] adj5 [therap$ or intervent$ or activit$ or psychotherap$ or interact$ or visit$ or program$]].ti,kw,ab.
(24) Exp equine-assisted therapy/
(25) 19 or 20 or 21 or 22 or 23 or 24
(26) Exp resilience, psychological/
(27) [Anxiet$ or anxious$ or worr$ or concern$ or apprehens$ or nervous$ or fear$ or distress$ or panic$ or neuros$ or apath$ or mood$ or dread$ or terror$ or phobia$ or irritable$].ti,ab,kw.
(28) Exp psychological distress/
(29) Exp stress, psychological/
(30) Exp stress disorder, traumatic/
(31) Exp stress, physiological/
(32) [Stress$ or burnout$ or burn-out$ or ‘burn out’].ti,ab,kw.
(33) [Depress$ or sad$ or sorr$ or unhapp$ or grie$ or lone$ or happ$ or dysthymia$].ti,kw,ab.
(34) [Internali? adj1 [disorder$ or symptom$ or behavio$]].ti,ab,kw.
(35) [Self$ adj/1 [esteem$ or accept$ or confiden$ or concept$]].ti,ab,kw.
(36) [[Emotion$ or mental$] adj/1 [health$ or illness$ or wellbeing or well-being or ‘well being]’ or cop$ or stress$ or burnout or burn-out or ‘burn out’ or resilien$]].ti,ab,kw
(37) Exp emotions/
(38) Exp depression/
(39) Exp mental health/
(40) Exp self concept/
(41) Exp mood disorders
(42) Exp anxiety disorders
(43) 26 or 27 or 28 or 29 or 30 or 31 or 32 or 33 or 34 or 35 or 36 or 37 or 38 or 39 or 40 or 41 or 42
(44) 18 and 25 and 43

**PsycINFO through OVID (from 1806 to April Week 3 2020):**

(1) [[animal$ or pet$ or dog$ or canine$ or hound$ or pooch$ or pup$ or cat$ or feline$ or kitt$ or equine$ or horse$ or hippo$ or pony$ or foal$ or riding$ or ‘guinea$ pig$’ or rabbit$ or bunn$ or ferret$ or hamster$ or rodent$ or mammal$ or bird$ or cow$ or pig$ or sheep$ or lamb$ or dolphin$ or aquatic$ or fish$ or marine$ or reptile$] adj5 [therap$ or intervent$ or activit$ or psychotherap$ or interact$ or visit$ or program$]].tw.
(2) “human$ animal$ interact$”.tw.
(3) “human$ animal$ bond$”.tw.
(4) Exp interspecies interaction/
(5) Exp animal assisted therapy/
(6) 1 or 2 or 3 or 4 or 5
(7) [college$ or universit$ or school$ or conservator$ or classroom$ or apprenticeship$ or faculty$].tw.
(8) [[educat$ or graduat$ or undergraduat$ or academ$ or junior$ or senior$ or postsecondary$ or “postsecondary$”] adj1 [school$ or colleg$ or universit$ or institut$ or setting$ or facult$ or establish$ or program$]].tw.
(9) [[seminar$ or lectur$] adj1 [room$ or theatre$]].tw.
(10) [high$ adj1 educat$].tw.
(11) Exp colleges/
(12) Exp schools/
(13) Exp classrooms/
(14) Exp apprenticeship/
(15) Exp higher education/
(16) Exp academic settings/
(17) Exp educational programs/
(18) Exp college environment/
(19) Exp educational degrees/
(20) Exp nursing education/
(21) Exp educational placement/
(22) Exp adult education/
(23) Exp academic environment/
(24) Exp campuses/
(25) [student$ or pupil$ or undergrad$ or postgrad$ or graduat$ or freshm?n* or sophomor$ or junior$ or senior$ or learner$ or scholar$ or apprentic$ or classmate$].tw.
(26) [[junior$ or senior$] adj1 year$].tw.
(27) Exp student/
(28) Exp classmates/
(29) 7 or 8 or 9 or 10 or 11 or 12 or 13 or 14 or 15 or 16 or 17 or 18 or 19 or 20 or 21 or 22 or 23 or 24 or 25 or 26 or 27 or 28
(30) [anxiet$ or anxious$ or worr$ or concern$ or apprehens$ or nervous$ or fear$ or distress$ or panic$ or neuros$ or apath$ or mood$ or dread$ or terror$ or phobia$ or irritable$].tw
(31) [depress$ or sad$ or sorr$ or unhapp$ or grie$ or lone$ or happ$ or dysthymia$].tw.
(32) [stress$ or burnout or burn-out or “burn out”].tw.
(33) [[emotion$ or mental$] adj1 [health$ or illness$ or wellbeing or well-being or “well-being” or cop$ or stress$ or burnout or burn-out or “burn-out” or resilien$]].tw.
(34) [internali? adj1 [disorder$ or symptom$ or behavio$]].tw.
(35) [self adj1 [esteem$ or accept$ or confiden$ or concept$]].tw.
(36) Exp emotions/
(37) Exp anxiety disorders/
(38) Exp neurosis/
(39) Exp irritability/
(40) Exp affective disorders/
(41) Exp well being/
(42) Exp anhedonia/
(43) Exp mental health/
(44) Exp emotional adjustment/
(45) Exp “resilience [psychological]”/
(46) Exp coping behaviour/
(47) Exp internalization/
(48) Exp self-esteem/
(49) Exp self-perception/
(50) Exp self-concept/
(51) Exp stress
(52) 30 or 31 or 32 or 33 or 34 or 35 or 36 or 37 or 38 or 39 or 40 or 41 or 42 or 43 or 44 or 45 or 46 or 47 or 48 or 49 or 50 0r 51
(53) 6 and 29 and 52

**Embase through OVID (from 1974 to 27 April 2020):**

(1) “human$ animal$ interact$”.ti,ab,kw.
(2) “human$ animal$ bond$”. ti,ab,kw.
(3) [[animal$ or pet$ or dog$ or canine$ or hound$ or pooch$ or pup$ or cat$ or feline$ or kitt$ or equine$ or horse$ or hippo$ or pony$ or foal$ or riding$ or ‘guinea$ pig$’ or rabbit$ or bunn$ or ferret$ or hamster$ or rodent$ or mammal$ or bird$ or cow$ or pig$ or sheep$ or lamb$ or dolphin$ or aquatic$ or fish$ or marine$ or reptile$] adj5 [therap$ or intervent$ or activit$ or psychotherap$ or interact$ or visit$ or program$]].ti,ab,kw.
(4) Exp animal assisted therapy/
(5) Exp human-animal bond/
(6) 1 or 2 or 3 or 4 or 5
(7) [colleg$ or universit$ or school$ or conservator$ or classroom$ or apprenticeship$ or facult$].ti,ab,kw.
(8) [[seminar$ or lectur$] adj1 [room$ or theatre$]].ti,ab,kw.
(9) [high$ adj1 educat$].ti.ab.kw.
(10) [[educat$ or graduat$ or undergraduat$ or academ$ or junior$ or senior$ or postsecondary$ or “post secondary$”] adj1 [school$ or colleg$ or universit$ or institut$ or setting$ or facult$ or establish$ or program$]].ti,ab,kw.
(11) Exp university/
(12) Exp college/
(13) Exp school/
(14) Exp school health service/
(15) Exp apprenticeship/
(16) Exp adult education/
(17) Exp doctoral education/
(18) Exp education program/
(19) Exp in service training/
(20) Exp medical education/
(21) Exp masters education/
(22) Exp paramedical education
(23) Exp postdoctoral education/
(24) Exp postgraduate education/
(25) Exp teacher training/
(26) 7 or 8 or 9 or 10 or 11 or 12 or 13 or 14 or 15 or 16 or 17 or 18 or 19 or 20 or 21 or 22 or 23 or 24 or 25
(27) [student$ or pupil$ or undergrad$ or postgrad$ or graduat$ or freshm?n* or sophomor$ or junior$ or senior$ or learner$ or scholar$ or apprentic$ or classmate$].ti,ab,kw.
(28) [[junior$ or senior$] adj1 year$].ti,ab,kw.
(29) Exp student/
(30) Exp graduate/
(31) 27 or 28 or 29 or 30
(32) [anxiet$ or anxious$ or worr$ or concern$ or apprehens$ or nervous$ or fear$ or distress$ or panic$ or neuros$ or apath$ or mood$ or dread$ or terror$ or phobia$ or irritable$].ti,ab,kw.
(33) [depress$ or sad$ or sorr$ or unhapp$ or grie$ or lone$ or happ$ or dysthymia$].ti,ab,kw.
(34) [stress$ or burnout or burn-out or “burn out”].ti,ab,kw.
(35) [[emotion$ or mental$] adj1 [health$ or illness$ or wellbeing or well-being or “well-being” or cop$ or stress$ or burnout or burn-out or “burn-out” or resilien$]].ti,ab,kw.
(36) [internali? adj1 [disorder$ or symptom$ or behavio$].ti,ab,kw.
(37) [self$ adj1 [esteem$ or accept$ or confiden$ or concept$]].ti,ab,kw
(38) Exp emotion/
(39) Exp anxiety disorder/
(40) Exp neurosis/
(41) Exp affect/
(42) Exp mood disorder/
(43) Exp stress/
(44) Exp temperament/
(45) Exp emotional disorder/
(46) Exp wellbeing/
(47) Exp coping behaviour/
(48) Exp psychological resilience/
(49) Exp mental health/
(50) Exp self concept/
(51) 32 or 33 or 34 or 35 or 36 or 37 or 38 or 39 or 40 or 41 or 42 or 43 or 44 or 45 or 46 or 47 or 48 or 49 or 50
(52) 26 or 31
(53) 6 and 51 and 52

**The Cochrane Central Register of Controlled Trials (CENTRAL) (from inception to 23 April 2020):**

(1) [Anxiet* or anxious* or worr* or concern* or apprehens* or nervous* or fear* or distress* or panic* or neuros* or apath* or mood* or dread* or terror* or phobia* or irritable*]:ti,ab,kw
(2) [Depress* or sad* or sorr* or unhapp* or grie* or lone* or happ* or dysthymia*]:ti.ab.kw
(3) [Stress* or burnout or burn-out or [burn NEXT out]]:ti,ab,kw
(4) [[Emotion* or mental*] NEAR/1 [health* or illness* or wellbeing or well-being or [well NEXT being] or cop* or stress* or burnout or burn-out or [burn NEXT out] or resilien*]]:ti,ab,kw
(5) [[Internali? NEAR/1 [disorder* or symptom* or behavio*]]]:ti,ab,kw
(6) [Self* NEAR/1 [esteem* or accept* or confiden* or concept*]]:ti,ab,kw
(7) MeSH descriptor: [Anxiety] explode all trees
(8) MeSH descriptor: [Anxiety Disorders] explode all trees
(9) MeSH descriptor: [Emotions] explode all trees
(10) MeSH descriptor: [Emotional Adjustment] explode all trees
(11) MeSH descriptor: [Expressed Emotion] explode all trees
(12) MeSH descriptor: [Mood Disorders] explode all trees
(13) MeSH descriptor: [Depression] explode all trees
(14) MeSH descriptor: [Trauma and Stressor Related Disorders] explode all trees
(15) MeSH descriptor: [Stress, Psychological] explode all trees
(16) MeSH descriptor: [Affective Symptoms] explode all trees
(17) MeSH descriptor: [Mental Health] explode all trees
(18) MeSH descriptor: [Resilience, Psychological] explode all trees
(19) MeSH descriptor: [Self Concept] explode all trees
(20) OR #1-#19
(21) [student* or pupil* or undergrad* or postgrad* or graduat* or freshm?n* or sophomor* or junior* or senior* or learner* or scholar* or apprentic* or classmate*]:ti,ab,kw
(22) [[junior* or senior*] NEAR/1 year*].ti,ab,kw
(23) MeSH descriptor: [Students] explode all trees
(24) OR #21-#23
(25) [colleg* or universit*or school* or conservator* or classroom* or apprenticeship* or facult*]:ti,ab,kw
(26) [[educat* or graduat* or undergraduat* or academ* or junior* or senior* or postsecondary* or [post NEXT secondary*]] NEAR/1 [school* or colleg* or universit* or institut* or setting* or facult* or establish* or program*]]:ti,ab,kw
(27) [[seminar* or lectur*] NEAR/1 [room* or theatre*]]:ti,ab,kw
(28) [high* NEAR/1 educat*]:ti.ab.kw
(29) MeSH descriptor: [College Fraternities and Sororities] explode all trees
(30) MeSH descriptor: [Universities] explode all trees
(31) MeSH descriptor: [Schools] explode all trees
(32) MeSH descriptor: [Clinical Clerkship] explode all trees
(33) MeSH descriptor: [Educational, nonprofessional] explode all trees
(34) MeSH descriptor: [Educational, professional] explode all trees
(35) MeSH descriptor: [Educational, predental] explode all trees
(36) MeSH descriptor: [Educational, premedical] explode all trees
(37) MeSH descriptor: [International Educational Exchange] explode all trees
(38) MeSH descriptor: [Inservice Training] explode all trees
(39) MeSH descriptor: [Academies and Institutes] explode all trees
(40) OR #25-39
(41) [human* NEXT animal* NEXT interact*].ti,ab,kw
(42) [human* NEXT animal* NEXT bond*].ti,ab,kw
(43) MeSH descriptor: [Bonding, Human-Pet] explode all trees
(44) MeSH descriptor: [Animal Assisted Therapy] explode all trees
(45) MeSH descriptor: [Equine-assisted therapy] explode all trees
(46) [[[animal* or pet* or dog* or canine* or hound* or pooch* or pup* or cat* or feline* or kitt* or equine* or horse* or hippo* or pony* or foal* or riding* or [guinea* NEAR pig*] or rabbit* or bunn* or ferret* or hamster* or rodent* or mammal* or bird* or cow* or pig* or sheep* or lamb* or dolphin* or aquatic* or fish* or marine* or reptile*] NEAR/5 [therap* or intervent* or activit* or psychotherap* or interact* or visit* or program*]]]:ti,ab,kw
(47) OR #41-#46
(48) #24 OR #40
(49) #47 AND #48 AND #20

**Grey literature through Advanced Google Search on 1 May 2020:**

Not signed in and as Google sorts by relevance first FOUR pages reviewed:

1st search: [“animal assisted therapy” OR “pet therapy”] AND [university OR college] AND [anxiety OR stress]
2nd search: [“human animal interaction” OR “animal assisted intervention”] AND [university OR college] AND [anxiety OR stress]
3rd search: [“animal assisted therapy” OR “pet therapy”] AND [undergraduate OR postgraduate] AND [anxiety OR stress]
4th search: [“human animal interaction” OR “animal assisted intervention”] AND [undergraduate OR postgraduate] AND [anxiety OR stress]

**PROSPERO International Prospective Register of Systematic Reviews on 4 May 2020:**

Search terms used:

Human animal interaction

Human animal bond

Animal therapy

Animal-assisted therapy

Animal-assisted activity

Animal-assisted intervention

Then all combinations of [pet, dog, canine, hound, pooch, puppy, cat, feline, kitty, equine, horse, hippo, pony, foal, riding, guinea pig, rabbit, bunny, ferret, hamster, rodent, mammal, bird, cow, pig, sheep, lamb, dolphin, fish, aquatic, marine, or reptile] with [therapy, intervention, activity, psychotherapy, interaction, visit, or program]

## Appendix B

**Additional decision rules for RoB2 assessment and vote counting**

**RoB2 Assessment:**

The decision rules were developed and applied to the RoB2 assessment to standardise the approach when information was lacking in the RoB2 guidance on how to approach. The specific domains and questions are taken directly from the RoB2 tool [67,68].

**“Domain 1: Risk of bias arising from the randomisation process**

1.3: Did baseline differences between intervention groups suggest a problem with the randomisation process?”

To allocate “No”/”Probably No” or “Yes”/”Probably Yes”: the baseline differences of the participants had to be split by each group to be able to compare. Additionally, the authors had to ensure it was clearly stated (either in the narrative or in Tables/Figures) that the characteristics were for the randomised participants. Providing only a statement regarding the characteristics for the whole group or a statement regarding statistical differences between the groups were not enough.

Otherwise, the grading of “No Information” was allocated.

**“Domain 2: Risk of bias due to deviations from the intended interventions (effect of assignment to intervention)**

2.6: Was an appropriate analysis used to estimate the effect of assignment to intervention?”

“Probably Yes” was allocated if all missing information was specified with reasons and the trialists did not state if deviations had occurred. The rationale was that the trialist may not state that deviations had not occurred due to word-count issues. Deviations are reportable to the relevant Institutional Review Boards (IRB) in accordance with ethical approval and trialists are expected to state deviations (if they occurred) according to good research practice.

“2.7: Was there potential for a substantial impact (on the result) of the failure to analyse participants in the group to which they were analysed?”

“Probably No” was allocated if excluded or missing data were less than 5% with appropriate reasons why.

**“Domain 3: Risk of bias due to missing outcome data**

3.1: Were data for this outcome available for all, or nearly all, participants randomised?”

For the allocation of “Yes” or “Probably Yes”: the authors had to clearly state the amount of missing data either in the narrative or in a Table/Figure and had to be less than 5%.

“No” was allocated if the missing data were more than 5%.

“No Information” was allocated if the amount of missing data was not clearly stated in either the narrative or Tables/Figures.

“3.3. Could missingness in the outcome depend on its true value?”

“No” was allocated if reasons were provided for the missing data and the reasons were not related to the outcome.

“Yes” was allocated if reasons were given and related to the outcome.

“No Information” was allocated if no reasons were provided.

If 3.4 required a response: “Is it likely that missingness in the outcome depended on its true value?”

“No information” was allocated if no reasons were provided for the missing data and the amount that were missing in each group was not clearly stated.

**“Domain 4: Risk of bias in measurement of the outcome:**

4.5: Is it likely that assessment of the outcome was influenced by knowledge of intervention received?”

Due to the nature of the intervention, even if the participants were blinded to the study’s purpose: “Yes” or “Probably Yes” was allocated.

**“Domain 5: Risk of bias in selection of the reported results:**

5.1 Were the data that produced the results in accordance with a pre-specified analysis plan that was finalised before unblinded outcomes data were available for analysis?”

For “Yes” to be allocated, the authors were required to publish their pre-specified analysis protocol in the public domain.

For “No” to be allocated, the authors had to both confirm pre-specified analysis protocol was not published and analysis was, indeed, not in accordance with an unpublished pre-specified plan.

“No Information” was allocated if no published pre-specified plan.

“5.2: Is the numerical result being assessed likely to have been selected, on the basis of the results from multiple eligible outcome (e.g., scales, definitions, time-points) within the outcome domain?”

“No” was allocated if the outcomes were measured at similar times in the same order with the same measures and same mode of administration for the groups involved.

“Yes” was allocated if the above elements varied.

“No information” was allocated if not enough information was given to assess adequately.

“5.3: Is the numerical result being assessed likely to have been selected, on the basis of the results, from multiple eligible analyses of the data?”

For “Yes” to be allocated, evidence was required to suggest multiple analyses had occurred either through the available information or through correspondence with the authors.

“No” was allocated if a pre-specified analysis protocol was published or the authors confirmed that the analysis was in accordance with a pre-specified unpublished analysis plan which was then made available for reviewers to examine.

“No Information” was allocated if the above criteria were not met.

**Vote counting:**

Table A1 summarises the decision criteria for both without and with a stressor. Specifically, in situations where a stressor was present, the expected response without the intervention was considered. For example, after a stressor (e.g., an experimental cognitive test) or before a known stressor (e.g., exam), the expectation is that anxiety scores are likely to be worse than baseline. For the intervention to have a beneficial effect when a stressor was present, three situations could occur:

(1) anxiety was worse after the intervention (as expected due to the stressor) but not by as much as the control group
(2) no change seen after the intervention, but anxiety was worse in the control group
(3) anxiety was better after the intervention and better than the control group

**Table A1:** Decision criteria to aid interpretation of vote counting.

Interpretation	Requirements
**Without a stressor**	
Beneficial	Post-intervention assessment shows improvement in scores (direction relative to the measure used) compared to pre-assessment and better than control
No	Post-intervention assessment: (1) shows no change in scores compared to pre-assessment or (2) improvement in scores (direction relative to the measure used) compared to pre-assessment but not as much as control
Detrimental	Post-intervention assessment shows worsening in scores (direction relative to the measure used) compared to pre-assessment and worse than control
**With a stressor** (either before the post-assessment or present during post-assessment, e.g., occurring prior to an exam)	
Beneficial	Post-intervention assessment shows: (1) worsening in scores (direction relative to the measure used) compared to pre-assessment as would be expected due to stressor but less than the control or (2) no change with the control scores being worse or (3) an improvement in scores which are better than the control
Detrimental	Post-intervention assessment shows worsening in scores (direction relative to the measure used) compared to pre-assessment as would be expected due to stressor and worse than control

## Appendix C

**Table A2:** Full text excluded studies with reasons (*n* = 122).

References of Excluded Studies from Full Manuscript Search	Reason Excluded
Adamle et al. [93]	wrong study design
Adams et al. [94]	wrong study design
Adams et al. [95]	wrong study design
Alonso [96]	criteria for inter-library loan not met
Anderson [97]	wrong outcome measures
Anonymous [98]	criteria for inter-library loan not met
Anonymous [99]	wrong study design
Ashton [100]	wrong study design
Baghain et al. [101]	wrong study design
Bajorek [102]	wrong outcomes
Barker et al. [103]	wrong outcomes
Barker et al. [104]	wrong outcomes
Barker et al. [105]	wrong study design
Barlow et al. [106]	wrong intervention
Basil et al. [107]	wrong study design
Behnke et al. [108]	wrong study design
Bell [2]	wrong study design
Beutler et al. [109]	wrong study design
Biery [110]	criteria for inter-library loan not met
Binfet et al. [111]	wrong study design
Binfet et al. [112]	wrong study design
Binfet et al. [113]	no comparator
Bjick [114]	wrong study design
Blender [115]	wrong intervention
Brelsford et al. [13]	wrong population
Broeyer et al. [116]	criteria for inter-library loan not met
Buttelmann et al. [117]	wrong study design
Chakales et al. [118]	wrong study design
Chramouleeswaran et al. [119]	wrong population
Cieslak [120]	wrong outcomes
ClinicalTrials.gov [121]	wrong intervention
ClinicalTrials.gov [122]	wrong outcomes
ClinicalTrials.gov [123]	wrong outcomes
ClinicalTrials.gov [124]	not included as trial still ongoing
Colarelli et al. [125]	wrong intervention
Coleman et al. [126]	wrong outcomes
Crago et al. [127]	wrong study design
Crossman et al. [6]	wrong study design
Crossman et al. [36]	wrong study design
Crump et al. [21]	wrong study design
Daltry et al. [34]	wrong study design
Delgado et al. [128]	wrong study design
Dell et al. [129]	wrong study design
Dhooper et al. [130]	wrong study design
Dluzynski [131]	wrong outcomes
Duffey T [132]	wrong study design
Flaherty [133]	wrong intervention
Folse et al. [134]	wrong study design
Frederick [135]	wrong population
Frederick et al. [136]	wrong population
Friedmann et al. [137]	wrong outcomes
Gonzalez-Ramirez et al. [138]	wrong outcomes
Goodkind et al. [139]	wrong population
Gress [140]	criteria for inter-library loan not met
Haggerty et al [141]	wrong study design
Hammer et al. [142]	wrong study design
Hemingway et al. [143]	wrong study design
Henry [144]	wrong intervention
House et al. [145]	wrong study design
Ishimura et al. [146]	wrong intervention
Jarolmen et al. [147]	wrong outcomes
Johnson [148]	wrong study design
King [149]	wrong study design
Kobayashi et al. [150]	wrong outcomes
Kronholz et al. [151]	wrong study design
Kuzara et al. [152]	wrong outcomes
Lacoff et al. [153]	wrong study design
Lauriente et al. [154]	wrong study design
Lephart et al. [155]	wrong study design
Linden [156]	criteria for inter-library loan not met
Litwiller et al. [157]	wrong study design
Machova et al. [158]	wrong study design
Malakoff [159]	wrong population
Manor [160]	criteria for inter-library loan not met
Marino [161]	wrong study design
Matsuura et al. [162]	wrong intervention
McArthur et al. [163]	wrong study design
McCrindle [164]	criteria for inter-library loan not met
McDonald et al. [165]	wrong intervention
Merritt [166]	criteria for inter-library loan not met
Morrison [15]	wrong study design
Morgan [167]	wrong study design
Muckle et al. [168]	wrong study design
Muellmann et al. [169]	wrong study design
Nocentini et al. [170]	wrong study design
Pendry et al. [171]	wrong outcomes
Pendry et al. [172]	wrong outcomes
Pendry et al. [173]	wrong outcomes
Pendry et al. [174]	wrong outcomes
Pendry et al. [175]	wrong intervention
Perry et al. [176]	wrong study design
Picard [177]	wrong intervention
Polking et al. [178]	wrong study design
Quinn et al. [179]	wrong study design
Ralston et al. [180]	wrong study design
Renne et al. [181]	wrong study design
Robino et al. [182]	wrong study design
Robson [183]	wrong study design
Rose [184]	wrong study design
Sanford [185]	wrong study design
Silas et al. [186]	wrong study design
Sola-Perkins [187]	wrong population
Stewart et al. [188]	wrong intervention
Stewart et al. [189]	wrong study design
Straatman et al. [190]	wrong intervention
Swan [191]	criteria for inter-library loan not met
Taylor et al. [192]	wrong study design
Thelwell [193]	wrong intervention
Thew [194]	wrong outcomes
Tobin [195]	wrong population
Tomaszewska et al. [196]	wrong study design
Trammell [197]	wrong outcomes
Turner et al. [198]	wrong study design
Voelpel et al. [199]	wrong study design
Walsh [200]	wrong study design
Wheeler et al. [201]	wrong intervention
Williams et al. [202]	wrong outcomes
Wilson [203]	not available due to COVID-19
Wilson [204]	not available due to COVID-19
Wood et al [205]	no comparator
Young [206]	wrong reporting of outcomes
Zents et al. [207]	wrong population

(Exclusion criteria were according to the methods. Additionally, if not correct study design and reported in a chapter, book or letter, was categorised as the wrong study design; wrong outcomes included wrong type of reporting outcome (e.g., anecdotal with no empirical data included) as well as wrong sequence of measuring outcomes; wrong intervention also included if stressor was not student-specific).

## Appendix D. Summary of the Theoretical Frameworks and Results

Theoretical frameworks:

For a study to be classed as clearly stating the theoretical framework that underpinned the included intervention, one of the following criteria was required:

- mechanism of action was stated and directly linked back to the intervention’s development before implementation; or
- proposal was offered for the intervention’s mechanism of action on the outcomes before the intervention was implemented; or
- mechanism of action was stated with a pre-specified assessment to distinguish the different co-interventions’ relative effects

Three of the included studies were assessed as articulating the theoretical framework according to the above [35,37,80]:

- Binfet [35] used Biophilia and Cobb’s Social Support Theory [24,26] stating that the study was “designed to both facilitate group interactions & human-animal relationship, factors theorized to contribute to stress reduction” ([35] p. 399).
- Grajfoner et al. [37] suggested that a benefit of dog-assisted interventions is “encouraging students to perceive counseling services as more accessible” & “therapy dogs represent a source of comfort, acceptance & de-stress” ([37] p. 2) with the study aimed to work out the relative influence of the dogs.
- Meola [80] considered experiential learning to address the structure proposed by Larson’s Social Cognitive Model of Counsellor Training (based on Banduras Social Cognitive Theory) as cited by Meola [80].

Seven of the included studies partially articulated the theoretical framework according to the above criteria [73,74,75,76,77,78,79]:

- Fiocco et al. [73] discussed the presence of therapy animal may act as a buffer of stress with no further expansion.
- Gebhart et al. [74] suggested distraction-focused techniques are “capable of creating some kind of break & may help students feel better” ([74] p. 3) with evidence that distraction techniques have reduced anxiety in other settings.
- Hall [75] applied Kolcaba’s Midrange Theory of Comfort [208] from patients to students. Suggested that students who reach transcendence (highest level of comfort) “would be empowered to rise above the challenges of nursing education” ([75] p. 203). Stating how & why dogs could help with achieving transcendence was not explicitly included.
- Hunt et al. [76] described the intervention of interest initially as a placebo control and in the discussion theorized the role of social support, community & sense of belonging.
- Shearer et al. [77] described the intervention of interest as an active control and in the discussion considered unconditional positive regard & social environment.
- Ward-Griffin et al. [78] suggested that therapy animals may act as a source of social support with no further expansion.
- Williams et al. [79] reported that therapy animals are trained to provide comfort, affection & can be calming with no further expansion.

One study [72] was assessed as not articulating the theoretical framework according to the above criteria:

- Banks et al. [72] discussed the benefits of canine interaction, for example the cognitive changes with reducing mind wandering & increasing sustained attention but did not explore how & why mental health outcomes would be improved.

Results:

Box A1 aids with interpretation of mean change scores and Table A3 provides summary of the results.

Box A1 Interpretation of the mean change scores (post-scores minus pre-scores) for each outcome measures; one example of the change score is presented with the inverse being true for the alternative option.

Anxiety as measured by:
STAI (regardless of subscale): negative change = anxiety decreases
HADS-anxiety subscale: negative change = anxiety decreases
Stress as measured by:
PSS: negative change = stress decreases
Depression as measured by:
BDI II: negative change = depression decreases
HADS-depression subscale: negative change = depression decreases
Mood/affect as measured by:
PANAS positive: negative change = positive mood decreases
PANAS negative: negative change = negative mood decreases
UMACL (depends on subscale): inference in paper was positive change = mood increases
Well-being as measured by:
WEMWBS: positive change = mental well-being increases
Subjective Happiness Scale: positive change = happiness increases
Total social support: positive change = total levels of support increases
SWLS (based on brackets): 5–9 = extremely dissatisfied; 15–19 = slightly dissatisfied; 20 = neutral; 21–25 = slightly satisfied; 26–30 = satisfied and as both groups in the same bracket that a negative score = satisfaction with life decreases

**Table A3:** Summary of results according to the hierarchy of comparators and time-points detailed in the methods. Mean change is post minus pre (unless specified) and when calculated by review SD cannot be provided. Vote counting is according to description in methods. NS = non-significant; NR = not reported. F/up = follow up.

Author & Year	Sample Size	Outcome	Findings with Effect Measures & Statistical Test Used by Authors	*p* Value	Evidence Strength	Vote Count	Conclusions
Banks [72] 2018	Randomised: *n* = 56 (*n* = 29 dog; *n* = 27 no-treatment control) Analysed: unclear	PANAS positive PANAS negative	Mean change after stressor; calculation by review. **Positive mood (PANAS positive):** Treatment: −4.51 No-treatment control: −3.48 **Negative mood (PANAS negative):** Treatment: 0.21 No-treatment control:−0.82 Mixed modal ANOVAs for change over time & if moderated by condition	**Mood:** PANAS positive: *p* > 0.05 (NS) for condition or time × condition PANAS negative: *p* > 0.05 (NS) for time, condition, time x condition	Overall weight: low	Mood (with stressor): PANAS positive & negative: Detrimental	Measurement occurred after a 10-min group free interaction single AAA session with a stressor applied before measurement (& sessions occurred during exam week). NS reduction in positive mood for both groups when condition or condition × time reviewed (treatment more than control). NS slight worsening of negative mood for treatment group. Where NS, no power calculation so unable to say if no true effect or if underpowered.
Binfet [35] 2017	Randomised: *n* = 163 Analysed: *n* = 155 (*n* = 81therapy dog; *n* = 74 no-treatment control e.g., studying)	PSS Sense of Belonging in School	Mean change (SD) **Stress (PSS):** Treatment: −0.17 (0.03) No-treatment control: 0.02 (0.04) **Well-being proxy (School belonging):** Treatment: 0.1 (0.03) No-treatment control: −0.05 (0.03) Used MANCOVA controlling for gender (inferred same approach with ANCOVA)	ANCOVA over time: treatment group vs. control Immediate: **Stress:** *p* < 0.001 **Well-being:** *p* = 0.002 (<0.05) 2-week f/up: NS	Overall weight: medium	Stress: Beneficial Well-being: Beneficial	Compared to control, statistical significance with improved scores were shown for both perceived stress (reduced) & school belonging (increased) for the treatment group (a 20-min group free interaction single AAT session). NS difference between the two groups at 2-week follow-up but no power calculation so unable to say if truly ‘no effect’ or if underpowered.
Fiocco [73] 2017	Randomised: *n* = 61 (*n* = 31 dog; *n* = 30 no-treatment control) Analysed: unclear	PANAS positive PANAS negative	Mean change inferred post minus pre after stressor (SD) **Positive mood (PANAS positive):** Treatment: −0.35 (6.66) No-treatment control: −4.37 (7.15) **Negative mood (PANAS negative):** Treatment: 2.29 (5.62) No-treatment control: 0.6 (6.1) ANCOVA controlling for baseline effect	**Mood:** PANAS positive *p* = 0.08 (NS) PANAS negative *p* = 0.61 (NS)	Overall weight: medium	Mood (with stressor): PANAS positive: Beneficial PANAS negative: Detrimental	For this individual free interaction 10-min single AAA session and subsequent stressor: Positive mood reduces (NS) for both but was worse for control. Negative mood was worse (NS) for both groups but more so for treatment group. Where NS, no power calculation so unable to say if no true effect or if underpowered.
Gebhart [74] 2019	Randomised: *n* = 72 Analysed: *n* = 57 **(*n* = 12 therapy dog;** ***n* = 15 no-treatment control (unstructured free hour);** *n* = 14 body percussion; *n* = 16 mandala painting)	STAI-S	Median change trends reported as relevant results only presented in graphs **Anxiety (STAI-S):** Normal day: Treatment: reduction No-treatment control: small increase Prior to exam (stressor): Treatment: small reduction No-treatment control: an increase Wilcoxon signed-rank tests	**Anxiety:** Normal day *p* < 0.01 for therapy dog; NS for control Exam day *p* = NS for both therapy dog & control	Overall weight: high	Anxiety (without stressor): Beneficial	On a normal day (after two structured group AAT interventions lasting 45–60 min of unknown time between sessions) a statistically significant reduction in anxiety was found in therapy dog group. On an exam day (after three structured group AAT interventions lasting 45–60 min of unknown time between sessions) NS difference in anxiety was found in both therapy dog & control group. Where NS, no power calculation so unable to say if no true effect or if underpowered.
Grajfoner [37] 2017	Randomised: Unclear; *n* = 132 recruited Analysed: *n* = 132 **(*n* = 45 dog with handler; *n* = 46 handler only (HO)** & *n* = 41 dog only (DO))	STAI WEMWBS UMACL	Mean change (SD NR) **Anxiety (STAI):** Treatment: −13.73 HO attention control: −2.02 **Well-being (WEMWBS):** Treatment: 2.36 HO attention control: −0.94 **Mood (UMACL):** Treatment: 2.62 HO attention control: −0.026 MANOVA across 3 groups with condition as between-participants factor & f/up Bonferroni tests	**Anxiety:** *p* < 0.001 **Well-being:** *p* < 0.001 **Mood:** NS (exact *p* value NR)	Overall weight: medium	Anxiety: Beneficial Well-being: Beneficial Mood: Beneficial	A free interaction 20-min single group AAA session demonstrated: (1) anxiety scores reduced for both dog with handler & control groups which was statistically significant in favour of the dog with handler group (2) statistically significant improvement in well-being in favour of the dog with handler group (3) NS significant improvement in mood (increased in dog with handler group with reduction in control) Where NS, no power calculation so unable to say if no true effect or if underpowered.
Hall [75] 2018	Randomised: Unclear; *n* = 109 recruited Analysed: *n* = 77 (*n* = 41 dog; *n* = 36 no-treatment control)	HADS	Mean change; calculation by review **Anxiety (HADS anxiety):** Treatment: −2.68 No-treatment control: −1.67 Independent *t*-test on pre- then post-scores by authors **Depression (HADS depression):** non-normal distribution & only given mean; Treatment: −0.93; No-treatment control: −1.55	**Anxiety:** *p* = 0.076 (NS) between pre-scores for control & dog. *p* = 0.008 between post-scores for control & dog **Depression:** NR (non-normal distribution)	Overall weight: medium	Anxiety: Beneficial Depression: No effect	A mixture of group/individual dog AAA sessions with free interaction & numerous opportunities to interact over 15–16 weeks, showed a statistically significant reduction in anxiety in favour of dogs on post-score (not controlled for pre-score; however, if evenly randomised can be appropriate [209]). Caution: depression scores not normally distributed (only means provided personal communication [81]). Appears to reduce in both groups: more in control group.
Hunt [76] 2018	Randomised: *n* = 119 Analysed: Unclear regarding final number analysed: (**study break with dog; no-treatment control;** mindfulness training alone; yoga alone; combined mindfulness & yoga training)	STAI-S PANAS positive PANAS negative	Mean change trends from baseline to after 1st session reported as relevant results only presented in graphs. Authors only report statistical results for after stressor. **Anxiety (STAI-S):** Treatment: reduction No-treatment control: an increase **Positive mood (PANAS positive):** Means NR **Negative mood (PANAS negative):** Treatment: reduction No-treatment control: no change Repeated measure ANOVA of condition across time & after each time-point; then pairwise comparisons	**Anxiety** (dog vs. control): No stressor: NS *p* = 0.07 by 4th session Stressor: anxiety higher *p* < 0.05 **Mood:** No stressor: PANAS positive: NS; PANAS negative: control worse mood over time than dog group (*p* ≤ 0.01); but NS by 4th session. Stressor: NS (*p* < 0.1)	Overall weight: medium	Anxiety: Beneficial Mood: PANAS positive: unable to assess PANAS negative: Beneficial	Group AAA sessions with free interaction of NR length once a week for 4 weeks with a dog (with games, icebreakers & snacks) demonstrated: (1) anxiety levels reduced for dog group compared to control (statistical significance only reported for 4th session = NS) (2) Control had statistically significant worse negative mood over time compared to dog group but not by 4th session After a stressor (1–3 weeks after interventions had finished), dog group had statistically significant worsening of anxiety levels & NS higher negative mood compared to control. Positive mood was NR. Where NS, no power calculation so unable to say if no true effect or if underpowered.
Meola [80] 2017	Randomised: *n* = 20 Analysed: *n* = 19 (unclear split between EALS & control)	STAI-S	Mean change; calculation by review **Anxiety (STAI):** Treatment: −0.16 No-treatment control: 0.09 Split plot MANOVA for pre- & post-test	**Anxiety:** *p* = 0.274 (NS)	Overall weight: medium	Anxiety: Beneficial	Individual 1-h structured AAT/AAE session with a horse demonstrated a small reduction in anxiety when measured up to 1 month after intervention (NS but underpowered).
Shearer [77] 2016	Randomised: *n* = 74 Analysed: Numbers analysed vary at each time-point: **‘destress with** **dog’; no-treatment control;** mindfulness meditation	STAI-S BDI II	Mean change; calculation by review **Anxiety (STAI-S):** No stressor: (1st session minus pre) Treatment: −15.25 No-treatment control: −3.5 With stressor (post minus pre): Treatment: 3.1 No-treatment control: 1.79 **Depression (BDI II):** interpreted as with stressor Treatment: −1.58; No-treatment control: −0.47 Repeated-measures ANOVA change over time, then planned comparisons (paired sample *t*-tests)	**Anxiety:** No stressor: control significantly different (*p* = NR) across time for anxiety when compared to dog group. Stressor: NS **Depression:** No stressor NR Stressor: NS	Overall weight: low	Anxiety: Beneficial Depression: Beneficial	Group free interaction AAA sessions with a dog including games & snacks lasting for 1 h/week for 4 weeks had statistically significant lower anxiety scores than control. After a stressor (1–2 weeks after interventions finished), anxiety levels increased in both groups but greater in treatment group (NS). Depression scores decreased in both groups with treatment group reducing more (NS) than control. Where NS, no power calculation so unable to say if no true effect or if underpowered.
Ward-Griffin [78] 2018	Randomised: *n* = 357 Analysed: *n* = 246 (*n* = 122 dog; *n* = 124 wait-list control)	PSS PANAS positive PANAS negative SWLS Subjective Happiness Scale Medical Outcomes Social Support	Mean change; calculated by review **Stress (PSS):** Treatment: −0.11; Wait-list control: 0.06 **Positive mood (PANAS positive):** Treatment: −0.52; Wait-list control:−0.44 **Negative mood (PANAS negative):** Treatment: −0.5; Wait-list control: −0.27 **Well-being proxies:** **Satisfaction with life (SWLS):** Treatment: −0.08; Wait-list control: 0.03 **Subjective Happiness Scale:** Treatment: −0.01; Wait-list control: −0.03 **Total Social Support:** Treatment: 0.1; Wait-list control: −0.03 Repeated measures ANOVA (effect of time x condition)	Effect of time x condition *p* = 0.007 negative mood; *p* = 0.031 stress; *p* = 0.032 total social support (gender as a fixed factor no significant interaction between condition, time & gender) *p* values otherwise NS	Overall weight: medium	Stress: Beneficial Mood positive: Detrimental Mood negative: Beneficial Well-being: SWLS: Detrimental Happiness: No effect Total Social Support: Beneficial	Group free interaction single AAA session with dogs lasting up to 90 min with outcomes measured up to 24 h after delivery, had statistically significant reduction in stress, negative mood & amelioration of total social support compared to control. Positive mood reduced for both groups & slightly more in treatment group (NS). Well-being: Satisfaction with Life: both groups were in the extremely dissatisfied category at baseline. Scores reduced in dog (NS) & did not change category. Happiness levels essentially did not change. Total social support significantly increased in treatment group. Where NS, no power calculation so unable to say if no true effect or if underpowered.
Williams [79] 2018	Randomised: *n* = 39 Analysed: *n* = 37 (*n* = 19 dog & *n* = 18 no-treatment control e.g., studying)	STAI-S&T	Mean change; calculation by review **Anxiety (STAI-S&T):** Treatment: 2.95 No-treatment control: 16.33 2-way mixed ANOVA, independent *t*-tests & Wilcoxon signed rank	**Anxiety:** *p* = 0.008 control had more anxiety than treatment group on exam day	Overall weight: medium	Anxiety (with stressor): Beneficial	For a 12-min inferred individual single AAA session delivered prior to an exam, anxiety levels increased for both groups with control having statistically significant higher levels of anxiety than dog.
